# Motor neglect associated with loss of action inhibition

**DOI:** 10.1136/jnnp.2007.140715

**Published:** 2008-11-10

**Authors:** E Coulthard, A Rudd, M Husain

**Affiliations:** 1 Institute of Neurology and National Hospital for Neurology and Neurosurgery, University College London, London, UK; 2 St Thomas’ Hospital and King’s College, London, UK

## Abstract

Motor neglect, underuse of one side of the body not explained by weakness or sensory impairment, is a common consequence of stroke that is surprisingly little understood. Behavioural and neuroanatomical hallmarks of the disorder are investigated. Using a masked prime task, it was shown that when patients with left motor neglect plan to move their left hand, irrelevant right limb motor programmes intrude, causing delay. Lesion analysis reveals that such asymmetry of motor programming occurs after infarcts of the right putamen and motor association areas. This demonstration of failure to inhibit ipsilesional limb motor plans suggests potential benefit from interventions that might act to restore balance in action planning.

Motor neglect, underuse of one side of the body not explained by weakness or sensory loss, occurs either in isolation or as part of the neglect syndrome,^1^ ^2^ complicating approximately a third of all stroke cases.^3^ Attempts to understand and successfully treat the condition have been hampered by diagnostic difficulty as it often coexists with hemiparesis.^4^ Neurophysiological evidence implicates failure to modulate inhibition of the primary motor cortex in patients with motor neglect, but the behavioural impact of these findings has never been investigated.^5^

Here we probe the inhibitory processes involved in motor neglect behaviourally using a masked prime task which offers an important window onto automatic inhibitory control.^6^ This paradigm has recently been deployed to probe deficits in Parkinson’s disease as well as focal lesions of supplementary motor areas.^7^ ^8^ In this task, prime arrow stimuli are presented for less than 50 ms and then masked (fig 1A). Normal observers are not able to report having seen the prime. However, the prime influences performance when a subsequent target arrow requires a response.

**Figure 1:**
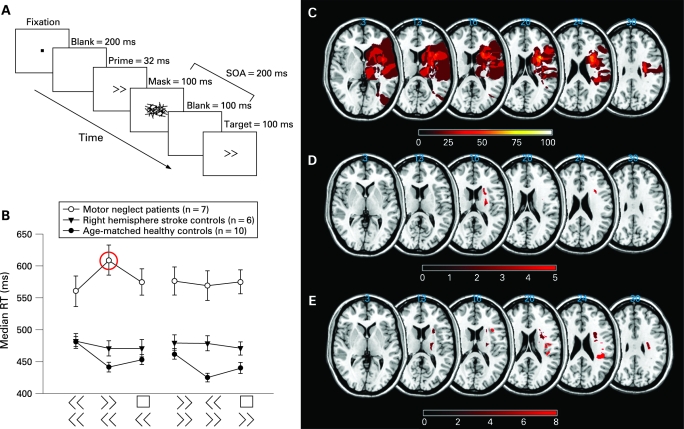
Paradigm, behavioural and lesion data. Subjects performed a masked prime task (A). Arrow stimuli subtended approximately 1.5×1°. Neutral primes comprised the arrows rearranged forming a square that carried no directional information (not shown). Twelve blocks of 24 stimuli contained six different trial types randomised with the constraint that each condition occurred the same number of times per block. Hands were covered during the experiment to prevent visual guidance of movement. Subjects were instructed to fixate the laptop display centrally and eye position was monitored by the experimenter. A practice session (<2 min) took place beforehand. Only patients with motor neglect showed significant reaction time delay when a right prime preceded a movement with the left hand (B, red circle). Hence right hand motor plans significantly intrude on left hand movement, but not vice versa, only in patients with motor neglect. Lesions were plotted using MRICro software (www.mricro.com) from either CT or MR. Lesion subtraction (patients with motor neglect minus those without) shows frontal white matter selectively affected in patients with motor neglect (C). (D) Brunner–Munzel statistic revealed that right putamen and subcortical white matter were significantly associated (z scores >4.47) with abnormal performance in the masked prime task using the left, but not the right, hand. (E) Severity of motor neglect is significantly associated (z >4.49) with damage at several discrete areas within the right hemisphere (including white matter near the putamen, inferior frontal gyrus, rolandic operculum and parietal supramarginal gyrus). RT, reaction time; SOA, stimulus onset asynchrony.

Unlike classical priming effects where similarity (congruence) between prime and target speeds response, if the interval or stimulus onset asynchrony (SOA) between mask and target is 100–200 ms, there is a paradoxical delay in reaction time when the prime and target are congruent —so-called negative compatibility effect. Thus motor programs evoked by the prime are inhibited if they do not continue to response initiation within ∼100 ms. Further, at these crucial SOAs (100–200 ms), there is facilitation when prime and target point are in different directions (incongruent condition).

Why does automatic inhibition of response plans occur? At any one time, we are confronted by a host of possible response alternatives to stimuli in the environment. In fact, remarkably, simply looking at an object may be sufficient to automatically and unconsciously activate motor plans to grasp it.^9^ Such “priming” is useful when we need to act quickly, but what if we do not want to perform the primed action? Clearly, flexible control over our actions requires the ability to inhibit action priming so that we can make other choices. Such flexible control may paradoxically occur automatically.

The negative compatibility effect is thought to probe brain mechanisms responsible for automatic inhibition of unwanted action plans. In the masked prime task, such unwanted, but automatic, response plans are generated by prime arrows. Exactly how these plans are classified as “unwanted” is unclear,^6^ but one possibility is that primed action plans are automatically inhibited if they do not develop further towards movement execution within 100–200 ms. Such inhibition allows alternative action plans to be made, permitting flexible control over behaviour. In fact, the mask prime task shows that at 100–200 ms after a prime (say right arrow), the alternative or incongruent movement (left response) is actually facilitated. Here we ask whether this is also the case in patients with motor neglect.

A possible analogy could be drawn between the negative compatibility effect and inhibition of return described in the visual attention domain, where orientation of attention back to areas recently visited is delayed.^10^ In normal individuals, inhibition of return has been suggested to ensure efficient visual search, preventing returns to previously searched locations. Lateralised breakdown of inhibition of return has been shown in patients with visual neglect.^11^ Here we investigate whether deficits in automatic action inhibition might underlie motor neglect.

We hypothesised that motor planning for the left arm is intruded on by conflicting movement plans for the right arm in right hemisphere patients with motor neglect. In other words, we expect that in patients with left motor neglect, irrelevant right primes will slow—not facilitate—movements of the left arm. In addition, we investigate which parts of the brain are associated with such asymmetric motor inhibition and motor neglect.

## METHODS

Seven patients with and six without motor neglect following right hemisphere stroke were examined along with 10 age matched healthy controls (table 1). All subjects gave informed consented with local ethics committee approval.

**Table 1:** Subject details

Description of symptoms	Age (y)	Time since stroke (months)	Bells cancellation score*	Line bisection score (mm rightward)	Personal neglect score†	Tactile neglect (N/Y)	Combined motor neglect score
Family noticed lack of movement on left side during daily activities	53	2	0	2	0	N	41.4
Difficulty walking due to “leaving left leg behind”. Finding arm hanging in uncomfortable positions and not being involved in activity particularly mobile phone use and eating	67	0.5	2	4	0	N	53.7
Absolutely no spontaneous activity in left arm unless prompted. Also great difficulty walking and wheelchair use dangerous as forgets to use one arm. Anosognosic	61	1	5	15	2 (left side)	Y	99.8
Difficulty with two handed activity, particularly eating and failure to move left hand noted by physiotherapist	69	1	0	3	0	N	22.8
Clumsiness reported by patient and friend. Limited spontaneous movement of hand noticed when writing	66	1	0	0	0	N	123.8
Complete failure to use the left hand except when prompted	78	2	6	18	0	Y	16.1
Tended to avoid using left hand except when prompted. Difficulty with make-up/washing reported by patient	36	2	0	−2	0	N	20.7

Motor neglect was assesses by uni- and bimanual motor neglect scores (summed to give the combined motor neglect score) to reflect the difference between the number of left and right handed movements in each condition: ((R − L) × 100) / (R + L) where R = number of right hand movements and L  = number of left hand movements. Fist opening is defined as >90° movement of the long axis of the second phalanx).

Non-motor neglect patients: performance range on combined motor neglect score −17 to 6.69 (average −1.71, SD 8.96); mean age 53.5 (range 44–71 years), none had significant weakness. It is important to note that there has been confusion in the literature about the term motor neglect, with some authors using it to describe other movement deficits following stroke, including directional impairments with the ipsilesional limb. However, many investigators now use the diagnostic label “motor neglect” to refer to the syndrome described here of underuse of the contralesional limb.

*Bells cancellation score (number of right-sided – left-sided cancellations).

†Personal neglect—modified Fluff test^20^ (eight post-it notes attached to the patient’s body while blindfolded; the patient was required to remove all post-it notes; score reflects number missed).

Motor neglect was diagnosed clinically on the basis of subjective complaint by the patient or caregiver of underuse of the left side in the absence of significant sensory loss, weakness, apraxia or ataxia. As it can be difficult to differentiate limb weakness from motor neglect, we specifically excluded patients with weakness on formal neurological examination at the time of experimental testing. Note that for our purposes we make the pragmatic distinction between motor neglect and paralysis on the basis of clinical examination. It is possible that severe motor neglect may manifest as paralysis but this study specifically did not include such patients. In addition, patients with motor neglect all displayed breakdown of alternating hand movements (ie, they were particularly impaired when asked to open one fist as they closed the other repeatedly).

We also developed an objective clinical measure of motor neglect severity based on the number of times each patient opened and closed his fist in 1 min using each hand separately versus both hands simultaneously with eyes closed, (table 1 and video. The video is available to view online).

Patients were positioned approximately 100 cm from a 15 inch Sony Vaio (PCG-5A1M) laptop screen where stimuli were presented centrally using Presentation (Albany, USA) software. A central prime stimulus was presented (32 ms) and subsequently masked, rendering the prime imperceptible (fig 1A). A speeded button press response with either the left or right hand (Cedrus button-box, San Pedro, USA) was required to the target arrow that followed the mask after 200 ms (SOA). To ensure the prime was successfully masked, all subjects were asked firstly to describe what they saw after the first block and then, at the end of the experiment, if they saw any arrows other than the ones following the hashed lines (mask), and none did.

There were 12 blocks of 24 stimuli each containing six different trial types randomised with the constraint that each trial type occurred the same number of times per block. Therefore, there were 48 data points for each trial type.

The Brunner-Munzel rank order test (www.sph.sc.edu/comd/rorden/mricron/) was used to investigation brain areas associated with abnormal behaviour. This is a relatively assumption free lesion analysis method in which continuous data are used to identify brain regions where damage correlates with impaired performance (fig 1D, E). This test was performed on lesion maps taken from the routine clinical imaging of all 13 stroke patients. At a given voxel, the average ranking on the behavioural measure in question of patients with lesions was compared with the ranking of those without damage at that voxel. In order to increase statistical power, only voxels where at least three subjects had a lesion were included in the analysis. This technique was used to identify areas associated firstly with abnormal performance in our experimental test and secondly with increasing severity of motor neglect (combined motor neglect score). Bonferroni correction was applied (post correction significance level of p<0.05).

## RESULTS

Our primary aim was to investigate the effect of incongruent primes, specifically selective intrusion of right hand motor plans (evoked by the prime) onto left hand movements (right prime followed by left target arrow = left incongruent condition). Secondly, we explored whether congruent primes resulted in reaction time slowing for left and right hand movements in patients with motor neglect, as expected in normal subjects.

As hypothesised, every patient with motor neglect was delayed when a right prime preceded a leftward movement (ie, in the left incongruent condition (fig 1B)). In contrast, age matched and stroke control groups showed the expected standard small benefit of incongruent primes bilaterally and no significant effect of the prime respectively (three way interaction: subject group × response side × prime type (F(4,40) = 4.772; p<0.005). Thus patients with motor neglect showed complete reversal of the incongruent prime interference pattern found in normal controls for left hand movements only.

Patients with motor neglect were slower than either control group even with the ipsilesional limb. Critically, however, there was no significant difference between the left and right hand overall response speeds in the motor neglect group. Therefore, the lateralised inhibitory deficits do not simply result from left-sided slowing. Generalised slowing is a well described finding in patients with right hemisphere stroke, potentially explained by attentional failure.^12^ Patients without motor neglect may have performed faster perhaps because either their lesions were relatively small or did not include regions involved in attentional processing.

What about the effect of congruent primes? Normal subjects had a standard negative compatibility effect (slower RT when prime and target arrow pointed in the same direction) with a significant average cost of congruence (F(2,18) = 32.539, p<0.001) (fig 1B). Neither stroke group showed any consistent or lateralised effect of congruent primes, suggesting that while automatic prime inhibition remained intact in some patients, it was lost in others, but that this did not correspond to the behavioural syndrome under consideration, motor neglect, as the variability occurred in both stroke groups.

Right−left incongruence costs were used for further analysis as they reflected lateralised difference in performance, controlling for factors such as age or slowness that could affect prime interference bilaterally.^7^ This experimental measure correlated significantly with the behavioural motor neglect severity score (Spearman’s rho 0.555, p<0.05) (fig S1 available online).

Brunner–Munzel analysis on the (right–left) incongruence cost for all 13 patients showed that right putamen and subcortical white matter were significantly more likely to be damaged in patients with intrusion of right hand motor plans onto leftward movements (fig 1D). Indeed, two of our patients with motor neglect with small strokes involving the putamen both demonstrated a cost of incongruence for left movements only, just like patients with larger lesions. In addition, close inspection of individual lesions revealed that all patients with motor neglect had lesions that involved the putamen, although the area of putamen involved differed between individuals.

We were also interested in how many patients with motor neglect had lesions involving the thalamus. Thalamotomy, a procedure used to treat dyskinesia, has previously been shown to result in motor neglect.^13^ Two of our patients had lesions that incorporated much of the thalamus and one patient had a lesion that encroached onto the ventral part of the thalamus.

Further lesion analysis looking at areas associated with behavioural motor neglect was performed using the Brunner–Munzel test, this time on the combined neglect severity score (table 1). Several small regions were significantly associated with motor neglect, including a region close to the putamen similar to that described above (fig 1E). Given the relatively small number of patients in the analysis and scattered areas associated with severe motor neglect, we feel inference from this lesion analysis should be cautious.

## DISCUSSION

This study reveals impairment in the ability to inhibit ipsilesional limb motor plans in motor neglect. Specifically, patients with left motor neglect fail to inhibit partially activated right motor plans (evoked by the prime), which then intrude abnormally on left hand action planning, slowing down initiation of movement with the left hand. Our experimental finding correlated with the severity of motor neglect, suggesting that such a mechanism might be causative in the manifestation of motor neglect.^14^ Consistent with our findings, one previous study revealed that monkeys with motor neglect following frontal lesions fail to inhibit their ipsilesional limb.^15^ Such intrusion of rightward incongruent primes onto leftward movement occurs despite intact automatic inhibition of congruent primes following masked prime presentation, at least in some patients. We note also that asymmetries in performance on the masked prime task may be the result of asymmetric interactions between disrupted inhibition and response to target arrows, not simply inhibition failure alone.^8^

Asymmetric intrusion of competing motor plans affecting left hand movements occurred particularly in patients with damage to the putamen and surrounding white matter, an area well connected to motor association and medial prefrontal regions. Interestingly, damage to the putamen has been associated with difficulty initiating movement in Parkinson’s disease, another condition where neglect-like phenomena occur in conjunction with inhibitory deficits.^16^ ^17^ Dopaminergic therapy alleviates, to some extent, the inhibitory abnormalities found in patients with Parkinson’s disease^18^ opening up the possibility of pharmacological intervention in motor neglect.

All patients also reported under-utilisation of the contralesional limb. However, as there is no standard clinical test for motor neglect, we developed a severity score for patients with motor neglect. This score reflects failure to move the contralesional limb during either bimanual or unimanual conditions, deficits sometimes referred to as motor extinction and motor impersistence, respectively. The aim of the score was to provide an objective, simple bedside clinical test that might suggest the presence and severity of motor neglect. However, it should be noted that abnormal performance could result from other disorders, including unilateral Parkinson’s disease, and so have to be interpreted appropriately in the clinical context.

When the severity score was compared with the lesion anatomy, several small motor association and subcortical brain regions significantly associated with increasing motor neglect severity were revealed, all consistent with previous anatomical descriptions of the condition.^1^ ^19^ ^20^ Transcranial magnetic stimulation and positron emission tomography activation studies also support the suggestion that while the primary motor cortex tends to be intact in patients with motor neglect, it is damage to the motor association areas that leads to the disorder.^5^ ^19^ We propose that when breakdown in part of this motor association network, including the putamen, causes intrusion of right movement plans (because of failure of inhibition) onto left-sided action planning, motor neglect results.

In summary, we have shown that lateralised inhibitory deficits are important in the genesis of motor neglect. We should highlight the fact that motor inhibition is likely to be a complex process involving interactions between multiple brain areas and we may have illuminated only one part of this network. Future work may investigate these complexities and in turn perhaps provide evidence for targeted therapeutic interventions to restore the inhibitory balance between the hemispheres, such as transcranial magnetic stimulation to the contralesional hemisphere, restraint therapy, used with some success in hemiparesis,^21^ or pharmacological intervention.
